# Activity of Trifluoperazine against Replicating, Non-Replicating and Drug Resistant *M. tuberculosis*

**DOI:** 10.1371/journal.pone.0044245

**Published:** 2012-08-31

**Authors:** Meeta J. Advani, Imran Siddiqui, Pawan Sharma, Hemalatha Reddy

**Affiliations:** 1 Department of Biochemistry, Sri Venkateswara College, University of Delhi South Campus, New Delhi, India; 2 Immunology Group, International Centre of Genetic Engineering and Biotechnology, ICGEB Campus, Aruna Asaf Ali Marg, New Delhi, India; International Center for Genetic Engineering and Biotechnology, India

## Abstract

Trifluoperazine, a knowm calmodulin antagonist, belongs to a class of phenothiazine compounds that have multiple sites of action in mycobacteria including lipid synthesis, DNA processes, protein synthesis and respiration. The objective of this study is to evaluate the potential of TFP to be used as a lead molecule for development of novel TB drugs by showing its efficacy on multiple drug resistant (MDR) *Mycobacterium tuberculosis* (*M.tb*) and non-replicating dormant *M.tb*. Wild type and MDR *M.tb* were treated with TFP under different growth conditions of stress like low pH, starvation, presence of nitric oxide and in THP-1 infection model. Perturbation in growth kinetics of bacilli at different concentrations of TFP was checked to determine the MIC of TFP for active as well as dormant bacilli. Results show that TFP is able to significantly reduce the actively replicating as well as non-replicating bacillary load. It has also shown inhibitory effect on the growth of MDR *M.tb*. TFP has shown enhanced activity against intracellular bacilli, presumably because phenothiazines are known to get accumulated in macrophages. This concentration was, otherwise, found to be non-toxic to macrophage *in vitro*. Our results show that TFP has the potential to be an effective killer of both actively growing and non-replicating bacilli including MDR TB. Further evaluation and in vivo studies with Trifluoperazine can finally help us know the feasibility of this compound to be used as either a lead compound for development of new TB drugs or as an adjunct in the current TB chemotherapy.

## Introduction

*M. tuberculosis* has been significantly contributing to the worldwide human infectious disease burden since long. The global TB crisis is further convoluted by the presence of MDR- and XDR-TB, being resistant to current antibiotics and hence hard to treat. It is a known fact that TB therapy has remained unchanged for nearly four decades now [1]. Moreover the existence of dormant TB in 90% of the TB affected individuals, poses a major hindrance in eradication of this dreadful disease. A number of promising new classes of compounds are currently in pipeline at various stages of discovery and clinical development [2]. An ideal therapy should consist of drugs that are active against the drug resistant varieties of *M.tb* they as well as can effectively target the sleeping bacilli lying within tubercular lesions.

Phenothiazines are known to have anti-mycobacterial activity for more than four decades. As the first line drugs against TB were able to effectively contain the disease, phenothiazines were not given much importance early on. Now with the advent of MDR strains of *M.tb,* compounds that can either be used directly or as adjuncts with the current drugs or may serve as lead compounds for the synthesis of new anti-TB drugs, are gaining importance. Trifluoperazine (TFP) is a calmodulin antagonist in eukaryotes [3] and has been used as an antipsychotic drug in neuroleptic patients [4]. Though many reports have indicated antimycobacterial activity of TFP [5]–[7], its exact mechanism of action is not yet clearly understood. Phenothiazines have been reported to affect the calcium-dependent ATPases, thereby reducing the amount of cellular energy required to maintain the active transport processes [8]–[11]. In mycobacteria, TFP has been shown to negatively affect processes like protein and lipid synthesis [7]. We have previously characterized the mycobacterial gene *Rv1211* as coding for a Calmodulin-like-Protein (CAMLP) in *M.tb,* with the ability to complex with calcium [12]. Our studies showed that this CAMLP-Ca2+ complex could stimulate heterologous targets like plant NAD Kinase and bovine brain phosphodiesterase (PDE). Knowing that TFP is a eukaryotic Calmodulin antagonist, we have checked its effect on *M.tb* CAMLP activities and have found it to be inhibitory [12].

In the present work, we demonstrate the efficacy of TFP in suppressing the growth/survival of two clinical isolates of MDR *M.tb* (JAL2287 and 1934) [13] *in vitro* as well as *ex vivo*. TFP also exerted lethal effect against stress induced (acidic, starvation and nitric oxide) persistent *M.tb*, thereby showing the potential to be effective against dormant TB.

## Materials and Methods

### Bacterial Strains and Culture Conditions

To prepare bacterial stocks, *Mycobacterium tuberculosis (H37Rv), M.tbJAL*2287 *and M.tb*1934 were grown to logarithmic phase (OD600∼0.6) in Middlebrook 7H9 broth supplemented with 10% albumin dextrose complex (ADC); 0.5% glycerol and 0.02% Tween 80 were also added to the media. Stocks were prepared by harvesting the bacteria, resuspending them in one-fifth volume of the original culture and storing in multiple aliquots at −80°C. The stocks were used as seed cultures for all the experiments. All bacterial cultures were incubated in a shaking incubator at 37°C at 170rpm.

### Determination of Minimum Inhibitory Concentration (MIC) of TFP for Mycobacteria

MIC of TFP for mycobacteria was determined as described previously [14]. Strains were grown in Middlebrook 7H9 broth supplemented with 10% ADC, 0.5% glycerol and 0.02% Tween 80 with an initial cell density ∼10^6^ cells/ml. TFP was added at final concentrations of 2.5, 5, 7.5 and 10 µg/ml. INH was added at concentration of 0.5 µg/ml as control for MDR strains. To the control, no drug was added. Growth of bacilli in absence and presence of TFP and INH was monitored by enumeration of colony forming units (CFUs), on days 0, 3, 6, and 9. Ten-fold serial dilutions were prepared with 7H9 broth and 50 µl of each dilution was plated on 7H11 agar supplemented with Middlebrook OADC enrichment. Plates were incubated at 37°C and 5% CO_2_. Colonies were counted after three weeks of incubation. This gives the number of colonies in the 50 µl aliquot applied to the plate. Total number of CFUs present was then determined. Graphs were plotted on log scale and growth inhibition at each concentration of TFP used was calculated.

### Determination of Minimum Bactericidal Concentration (MBC) of TFP for *Mycobacterium tuberculosis* (*H37Rv*)

Minimum Bactericidal Concentration of TFP for *M. tuberculosis (H37Rv)* was determined as described elsewhere [15], with slight modifications. Briefly, bacilli were grown in 250 ml of the complete Middlebrook 7H9 broth until mid-log phase was obtained (OD600nm∼0.5). At this stage the cells were pelleted down at 5000rpm for 30 minutes at room temperature and re-suspended in 28 ml of 7H9 broth. 7 ml of this suspension was then dispensed into 4 Erlenmeyer flasks each containing 20 ml 7H9 broth supplemented with 3 ml ADC, followed by addition of TFP at final concentrations of 0, 5, 10 and 20 µg/ml. The cultures were allowed to shake at 170rpm, 37°C. To monitor the effect of TFP on growth and survival of bacteria, enumeration of CFUs was done on days 0, 3, 5, 7 and 10 post drug treatment. Ten-fold serial dilutions were prepared with 7H9 broth and 50 µl of each dilution was plated on 7H11 agar supplemented with Middlebrook OADC enrichment. Plates were incubated for 21 days at 37°C. Colonies were counted on day 21. Total number of CFUs was determined as described above to plot the graph, and the percentage inhibition at each concentration of TFP used was calculated.

### Susceptibility of Stress Induced Persistent *M.tb* Cultures to Trifluoperazine

#### Acidic

The effect of TFP against *M.tuberculosis (H37Rv)* was tested at pH6.8 and pH5.5 in complete Middlebrook 7H9 broth as described previously for *M. avium* [16]. 7H9 broth (pH 6.8) was adjusted to pH 5.5 using HCl. *M.tuberculosis (H37Rv)* was cultured in 30 ml Middlebrook 7H9 broth (pH 5.5 and pH 6.8*)* supplemented with 10% AD*C* with initial density of 5×10^6^ cells/ml. To evaluate the activity of TFP at pH 5.5, it was added at final concentration of 5 and 10 µg/ml. Controls were kept without TFP at both pH 6.8 and pH 5.5, to compare the growth of bacteria at these pH without drug. Growth of mycobacteria in the two media in the presence and absence of TFP was monitored by enumerating CFUs on days 0, 5, 7, 8, and 10 as described previously.

#### Starvation

To determine the effect of trifluoperazine against starved *M. tuberculosis (H37Rv), Betts et al* model was adopted [17]. Briefly, bacilli were grown in 250 ml of complete Middlebrook 7H9 broth till mid log phase (OD_600nm_∼0.5). At this point the culture was dispensed into five 50 ml centrifuge tubes each receiving 50 ml of the suspension. The culture was centrifuged at 5000 rpm for 30 minutes. The pellet obtained was washed with sterile Phosphate Buffer Saline (PBS) twice. Finally, pellet in each tube was resuspended in 7 ml PBS and pooled together. 5 flasks containing 45 ml PBS were each inoculated with 5 ml of the suspension. Two flasks containing 45 ml complete Middlebrook 7H9 were also inoculated with 5 ml of the suspension. The bacterial suspension was then incubated at 37°C in static incubator for 6 weeks. CFUs were enumerated on days 0, 21 and 42. On day 42, cultures were treated with TFP (10 ug/ml) and INH (0.25 ug/ml) in duplicates separately. CFUs were enumerated as before on days 1, 3 and 5 post drug treatment. All plates were incubated for 21 days at 37°C. Number of CFUs were calculated as described previously.

#### Nitric Oxide

Nitric oxide has been known to be key to arrest of *M. tuberculosis* growth in mice. This phenomenon has been modeled *in vitro* by treatment of *M. tuberculosis* with diethylenetriamine- nitric oxide adduct (DETA-NO), a generator of nitric oxide. DETA NO decomposes and releases NO in the culture medium causing bacteriostasis. To check the efficacy of TFP in killing of NO induced NRP *M.tb*, mid log phase *M.tb* cultures were subjected to NO stress by treating with 50 uM DETA NO for 16 hrs. Attainment of bacteriostasis was confirmed by enumerating CFU at week 1, 2 and 3. After 16 hr treatment culture was treated TFP (5 ug/ml). Growth kinetics of treated and untreated bacilli in presence of TFP was monitored by enumerating CFU at days 1, 7, 14 and 21 post treatment.

### Effect of TFP on Intracellular Growth Kinetics of *M.tb*

#### THP-1 cell model

Activated THP-1 cells model was adopted for the assessment of intracellular growth perturbation of *M.tuberculosis (H37Rv, JAL2287 and 1934)* in presence of TFP [18]. Briefly, THP-1 cells (American Type Culture Collection) were subcultured every third day for an initial density of 1×10^6^ cells/ml. Cell viability was assessed by trypan blue exclusion. THP-1 cells were differentiated into adherent, well spread macrophages by the addition of 20 nM phorbol myristate acetate (PMA) and maintained at 37°C, 5% CO_2_, to adhere overnight, prior to utilization in experiments. The adherent cells were washed with fresh RPMI1640 prior to the addition of *M. tuberculosis* at an MOI of 10∶1. Plates containing infected cells were then centrifuged at 700 g for 5 mins followed by 4 h of stationary incubation at 37°C, 5% CO_2_. After 4 hrs, bacilli not associated with the cells were removed by washing of the monolayers with RPMI1640. Infected monolayers that were not immediately processed to assess uptake (day zero) were supplemented with fresh RPMI and re-incubated at 37°C, 5% CO_2_ for a period of 3 days. TFP and INH were added at various concentrations (1, 2.5, and 5 ug/ml for TFP and 0.25, 0.5 and 1 ug/ml for INH). To controls no drug was added. Bacterial growth and survival in presence and absence of TFP and/or INH was monitored by lysing macrophages at day 1, 2 and 3, using lysis buffer (0.05% Triton X-100 in normal saline) and vortexing. The released bacteria were plated on 7H11 agar plates after serial dilution and CFUs were enumerated after 3-week incubation at 37°C. Macrophage viability was monitored by trypan blue exclusion at each time point.

#### Peripheral blood mononuclear cells model

Peripheral blood mononuclear cells (PBMCs) were isolated from 20 ml human blood collected from a healthy donor. Blood sample was diluted in 40 ml of 0.15 M PBS. PBMCs were isolated by centrifugation with Ficoll-Hypaque (Histopaque 1077, Sigma) at 1800 rpm for 30 minutes at room temperature. PBMCs were washed twice with PBS and resuspended in serum free RPMI 1640 medium containing 25 mM HEPES (*N*-2-hydroxyethylpiperazine- *N*9-2-ethanesulfonic acid) (Gibco Laboratories, Grand Island, N.Y.) supplemented with 2 mM L-glutamine. Cells were dispensed in two separate tissue culture petriplates and incubated for 2 h at 37°C under humidified 5% CO_2_ for panning. After 2 h the non-adherent cells were aspirated and complete RPMI 1640 with 10% heat inactivated FCS was dispensed in the perti-plates containing the adhered monocytes. Monocytes were allowed to differentiate into macrophages by incubating for 5 to 6 days and replacing media twice in between. Finally human monocyte dervived macrophages (MDMs) were seeded at density of 1×10^4^ per well in a 96 well plate. For infection freshly cultured *M.tb*H37Rv and *M.tb*JAL 2287 were brought to a density of 1×10^6^ bacilli/ml. Cells were pelleted and resuspended in equal volume of RPMI 1640. 100 µl of the above suspension was added in each well containing adhered macrophages to make an MOI of 10∶1. Infection protocol followed was same as given for THP-1 model. TFP was added at concentrations of 1, 2.5 and 5 µg/ml in duplicates. Control with no drug was also kept for each strain.

### Analysis of MDM Viability

MDM viability following treatment with various concentrations of TFP was measured by using MTT method. For each MTT assay 4×10^4^ differentiated human macrophages were plated on 96 well tissue culture plate using media as described previously and allowed to adhere for 2 hours. Macrophages were incubated with and without TFP for a period of 3 days. MTT was added at 0, 24, 48 and 72 hour. MDMs were allowed to metabolize MTT for 2 hours, after which media was removed and cells were treated with 100% dimethyl sulfoxide to lyse the cells and dissolve formazan crystals. Absorbance of the lysates was measured at 560 nm. Blank wells were subtracted from each duplicate sample and samples were averaged.

## Results

### Trifluoperazine Inhibits Growth of MDR *M.tb in vitro* and *ex vivo*

**Figure 1:**
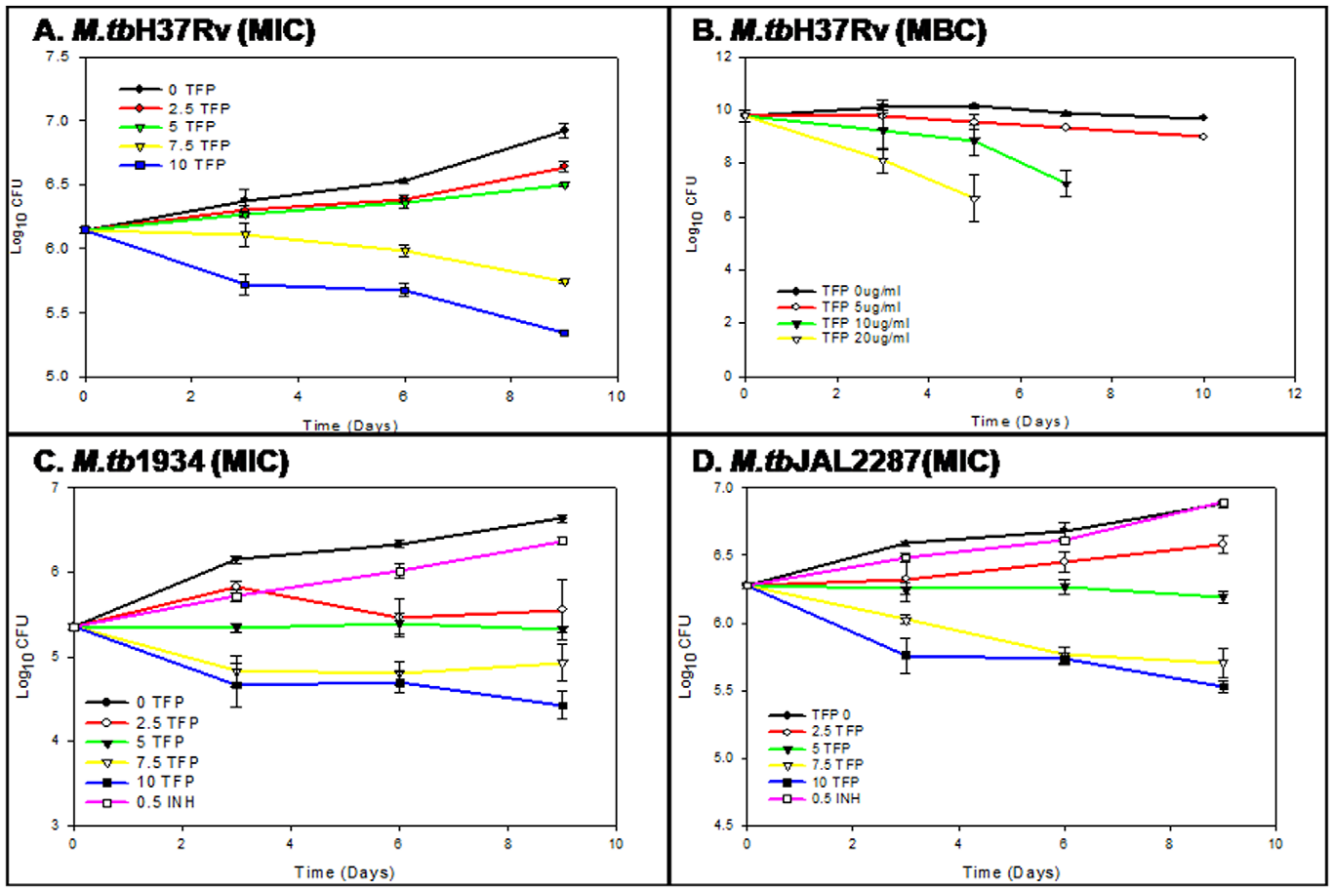
In vitro susceptibility of multi-drug resistant strains of *M.tb* to trifluoperazine. Perturbation in growth kinetics of MDR and WT *M.tb* in presence of different concentrations of TFP was monitored by enumerating CFUs on days 0, 3, 6 and 9 as described in materials and methods to determine MIC and MBC of TFP for *M.tb*. The figure shows the growth curve of (**a**) M.tbH37Rv (MIC), (**b**) M.tbH37Rv (MBC), (**c**) M.tb1934 and (**d**) M.tbJAL2287 in presence of 0, 5, 10 and 20 µg/ml of TFP. For MDR strains 0.5 µg/ml of INH was used as control. Values represent means ± standard errors of the means of duplicates.

Many groups have reported the MICs of various phenothiazines against mycobacteria in the range of 4–32 µg/ml [19]. In the present study, efficacy of TFP against MDR strains of *M.tb* was assessed by determining its MIC for two of the MDR *M.tb* clinical isolates - JAL2287 and 1934. MIC was defined as the lowest concentration of TFP used, that inhibits the growth of bacilli by minimum 1.2 Log_10_ units as compared to control (cultures without TFP). TFP was used at final concentrations of 0, 2.5, 5, 7.5 and 10 µg/ml and growth inhibition of *M.tb(H37Rv)*, *M.tb*(JAL2287) and *M.tb*(1934) was determined spectrophotometrically (OD_600nm_) (data not shown) and by CFU assay (Figure 1). The results in terms of decrease in Log_10_CFU for each strain are given in table 1. As can be seen, at 10 µg/ml, TFP was able to completely inhibit the growth and survival of wild type *M.tb* giving a decrease of 1.58 log_10_ units. TFP enhanced inhibition of growth of MDR strains with lower MICs of 7.5 and 5 µg/ml for *M.tb*JAL2287 and *M.tb*1934 respectively. Antimicrobials are usually regarded as bactericidal if the Minimum Bactericidal Concentration (MBC) is no more than four times the MIC [20]. At 10 µg/ml TFP was found to completely sterilize a load of ∼4×10^9^ bacilli within 1 week. While the MBC:MIC ratio of 1 for M.tbH37Rv demonstrates its potent bactericidal attribute, its lower MICs for drug resistant varieties of *M.tb* suggests that this compound can also be used to develop drugs for effective management of the fast expanding pool of drug resistant *M.tb*.

**Table 1:** MIC of TFP for WT and MDR *M.tb.*

Strain	Reduction in growth (Log_10_)				MIC (µg/ml)
	TFP (µg/ml)				
	2.5	5	7.5	10	
*M.tb* H37Rv	0.283	0.424	1.18	1.58	7.5
*M.tb*JAL2287	0.3	0.7	1.2	1.35	7.5
*M.tb*1934	1.08	1.31	1.7	2.21	2.5

Table gives values of log scale reduction in growth of different strains of mycobacteria in presence of various concentrations of TFP. Concentration of TFP giving a minimum 1 Log _10_ reduction was considered as MIC for the strain.

MIC – Minimum Inhibitory Concentration.

**Figure 2:**
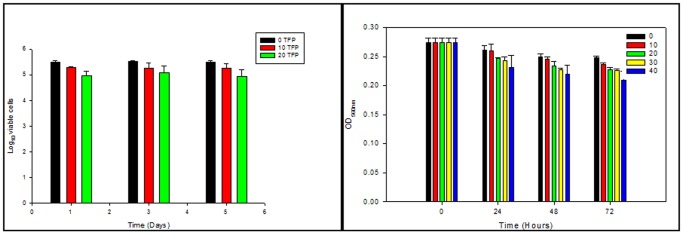
Viability of macrophages in presence of trifluoperazine. (**a**)THP-1 cells were cultured in RPMI 1640 medium supplemented with 10% FCS at 37°C, 5% CO_2_ for a period of 5 days. Dose dependent response of TFP on THP-1 cells viability was determined by culturing THP-1 cells in different concentration of TFP (0, 10 and 20 µg/ml). Viability of cells was determined by trypan blue exclusion method at days 1, 2, 3 and 5. (**b**) Monocyte derived macrophages were treated with different concentrations of TFP (0, 10, 20, 30 and 40 µg/ml). Cell viability was checked by MTT assays after 24, 48 and 72 hours of treatment. Values represent means ± standard errors of the means of duplicates.

Studies on *in vitro* antimycobacterial activity of any new molecule may lead to false inferences unless assessed under conditions resembling those *in vivo*. Since TFP significantly contributed to sterilization of MDR strains *in vitro,* we exploited the THP-1 cells *intramacrophage and MDMs ex vivo* model to study the effect of TFP on intracellular growth kinetics of these strains. Phenothiazines are known to get accumulated within macrophages and thus show enhanced activity against intracellular bacilli [21]. In the present study, the intracellular activity of TFP was determined by treating *M.tb* (wild type and MDR) infected THP-1cells and MDMs (MOI –10∶1) with 1, 2.5, and 5 µg/ml of TFP. Dose dependent response of TFP on THP-1 cells viability was also assessed by culturing THP-1 cells in presence of 0, 10 and 20 µg/ml of TFP for a period of 5 days. Percentage viability of THP-1 cells on days 3 and 5 was found to be 80% and 75% respectively as observed by trypan blue exclusion method (Figure 2A). To check whether TFP affected the viability of MDMs, MTT assays was performed. This method measures cell vialbility by assessing the presence of active mitochondrial dehydrogenases that convert MTT into water-insoluble, purple formazan crystals. As shown in figure 2B, MDMs showed 95%, 92%, 92% and 84% viability post 72 hrs of treatment with 10, 20, 30 and 40 µg/ml of TFP respectively.

**Figure 3:**
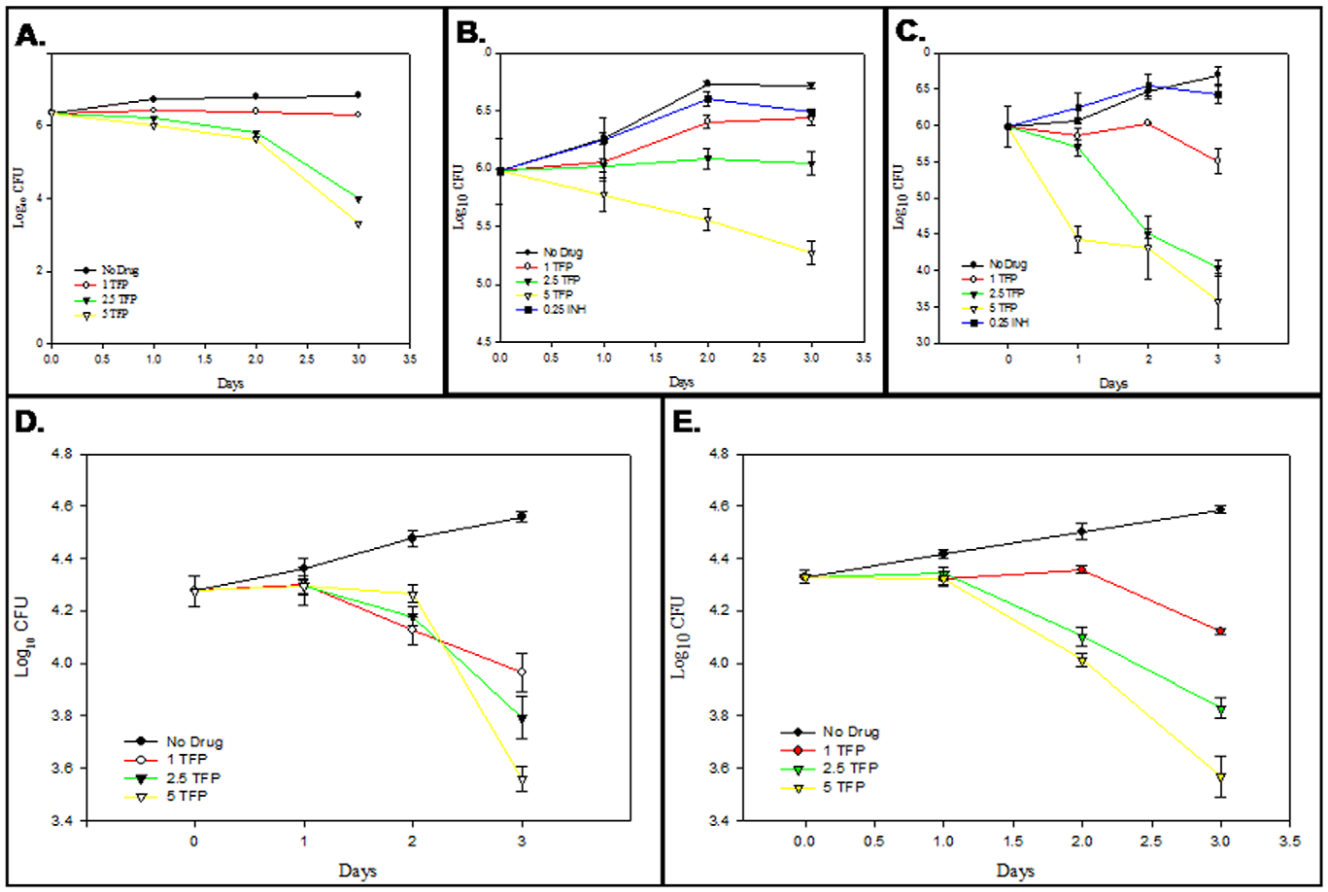
Ex vivo susceptibility of multi-drug resistant strains of *M.tb* to trifluoperazine. THP-1 cells and MDMs infected with *M.tb* at an MOI of 10∶1were treated with different concentrations (0, 1, 2.5 and 5 µg/ml) in duplicates as described in materials and methods. Post 1, 2 and 3 days of infection, cells were lysed and plated on 7H11 to enumerate CFUs. Graphs depict intracellular growth perturbation of (**a**) *M.tb*H37Rv in THP-1 cells, (**b**) *M.tb*JAL2287in THP-1 cells, (**c**) *M.tb*1934 in THP-1 cells, (**d**) *M.tb*H37Rv in MDMs and (**e**) *M.tb*JAL2287 in MDMs in presence of various concentrations of TFP. Values represent means ± standard errors of the means of duplicates.

Effect of TFP on intracellular growth and survival of *M.tb* was monitored by lysing THP-1 cells and MDMs at different time points and enumerating *M.tb* CFU. Inhibition in intracellular bacilli growth as observed on day 3 was calculated. For the wild type strain *M.tb*H37Rv, TFP showed a reduction of 0.53, 2.82 and 3.5 log_10_ units in THP-1 cells and 0.6, 0.8 and 1 log_10_ units in MDMs in number of CFU at concentrations of 1, 2.5, and 5 µg/ml respectively as compared to that in the control (Figure 3A and 3D). In case of MDR strains this reduction amounted to 0.28, 0.67 and 1.44 log_10_ units in THP-1 cells and 0.5, 0.75 and 1 log_10_ units for *M.tb*JAL2287 (Figure 3B and 3E) and 1.18, 2.65 and 3.11 log_10_ units for *M.tb*1934(Figure 3C) at 1, 2.5, and 5 µg/ml respectively. Since both strains are known to be resistant to INH [13], it was used as a control at concentration equal to its *in vitro* MIC for *M.tb*H37Rv (0.25 µg/ml) giving a reduction of 0.23 and 0.14 log_10_ units for *M.tb*JAL2287 and *M.tb*1934 respectively (Figure 3B and 3C). Results clearly demonstrate that for *M.tb*H37Rv TFP exhibits 10 fold greater reduction in number of CFU in macrophage model when compared to that in broth cultures. In case of multi drug resistant strains this increase in reduction amounted to approx. 2.4 fold for each of the strain. However both the strains showed resistance to INH in the macrophage model.

### TFP is Lethal Against Mycobacteria at Low pH

**Figure 4:**
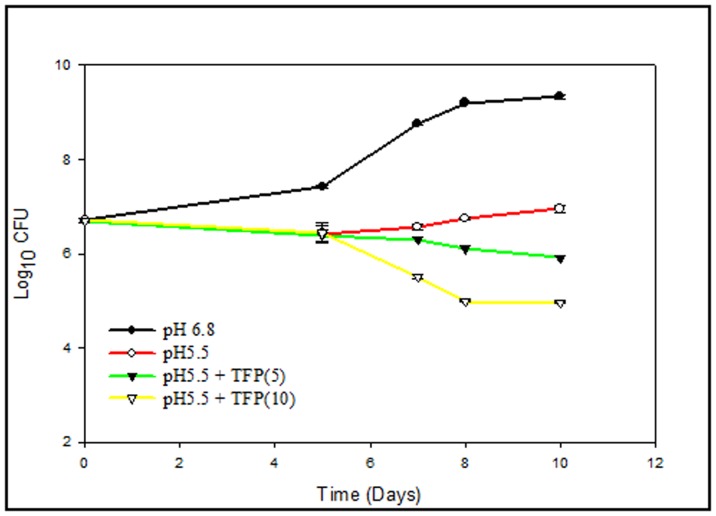
Effect of pH on lethality of TFP against *Mycobacterium tuberculosis (H37Rv).* The effect of TFP against *mycobacteria* was tested at pH 6.8 and pH 5.5 in Middlebrook 7H9 broth. 7H9 broth (pH 6.8) was adjusted to pH 5.5 using HCl. TFP was added at final concentration 10 µg/ml. Controls were kept without TFP at both pH 6.8 and pH 5.5. Growth of bacilli in the two media in the presence and absence of TFP was monitored by enumerating CFUs at various time points as described in materials and methods. Graph depicts effect of pH on activity of TFP against *M.tuberculosis (H37Rv)*. Values represent means ± standard errors of the means of duplicates.

*M.tb* is an intracellular organism that resides and proliferates predominantly in the macrophages. The pH of the intracellular environment within macrophages is known to be in the range of 5.0–5.5 [22]–[23]. Therefore, knowing the success of *M.tb* as an intracellular pathogen, it seems pertinent to test the efficacy and stability of a potent anti-TB agent under such conditions of stress. We have selected pH5.5, to mimic the intraphagosomal environment in broth cultures and checked the anti-mycobacterial action of TFP. *M.tb* is known to have certain virulent factors that are able to modulate host cell signaling such that phagosome maturation and its acidification is inhibited [24]–[26]. Phenothiazines are potent inhibitors of Ca2+-dependent ATPase mediated efflux of K^+^ and Ca^2+^ from phagolysosome, thus leading to its acidification and putting the bacilli under strain [20]. However, adaptation of *M.tb* and *M. avium* in these stressful acidified macrophages has been reported [27]. We have checked the influence of pH on growth and survival of pathogenic as well as non-pathogenic bacteria. *M. smegmatis* was not able survive well under the acidic environment (data not shown). *M.tb* culture at pH5.5 exhibited a prolonged lag phase as compared to culture at pH6.8. As can be seen in Figure 4, decrease in pH from 6.8 to 5.5 showed inhibitory effect on the growth of *M.tb*. The increase in number of CFUs from day 0 to day 10 post inoculations at pH6.8 was found to be 1.635 whereas that at pH5.5 was only 0.335 log_10_ units. Although *M.tb* was not able to proliferate well at pH5.5, it was able to survive in the acidic environment as evident from no loss in number of CFUs. This can be speculated as the time taken by the bacilli to adapt to the mildly acidic milieu. A number of genes have been reported to be differentially regulated in *M.tb* during such mildly acidic conditions [28]–[29], signifying the presence of an adaption mechanism. Regardless of the pH, TFP was effective in inhibiting growth and survival of *M.tb* at concentrations of 5 and 10 µg/ml. The inhibition was found to be 3.42 and 4.3 log_10_ units at for 5 and 10 µg/ml respectively at pH5.5. Though the enhancement in killing action of TFP at pH5.5 might depict the influence of multiple stresses of pH and drug on bacilli, nevertheless, it speaks for the stability and effectiveness of TFP in inhibiting the bacilli growth under acidic conditions.

### TFP Kills Starvation-induced Persistent *M.tb*

**Figure 5:**
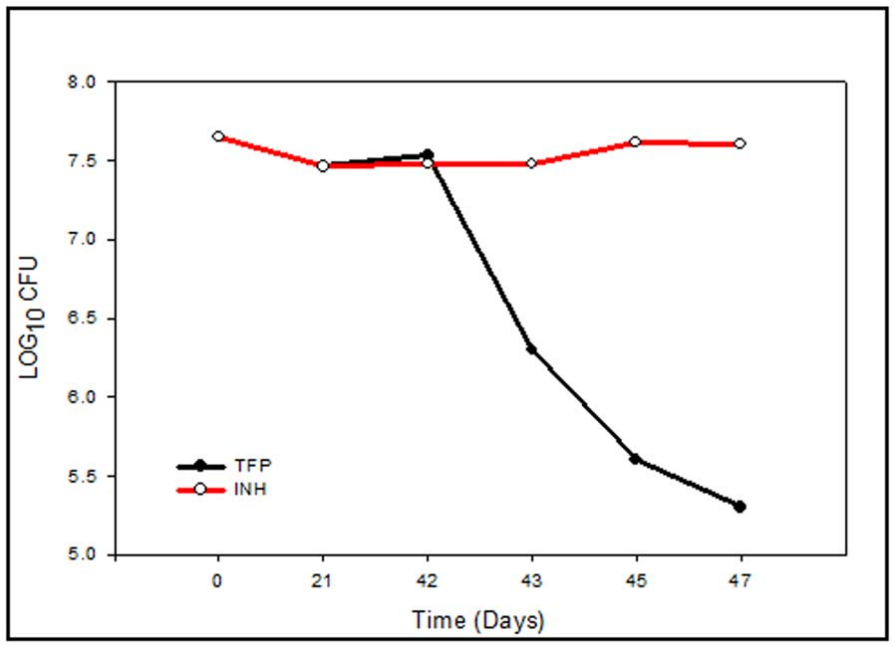
Susceptibility of starved *M.tuberculosis (H37Rv)* to TFP *in vitro).* Mid log phase *M.tb* cultures were starved in PBS for 6 weeks to induce bacteriostasis. Attainment of bacteriostasis was confirmed by enumerating CFU at week 0, 3 and 6. Starved cultures were treated with INH and TFP at their MIC separately in duplicates. Survival of bacilli in presence of drugs was monitored by enumerating CFUs on days 1, 3, and 5 post drug treatment. Graph depicts the effect of TFP and INH on starvation induced bacteriostatic *M.tb* cultures. Values represent means ± standard errors of the means of duplicates.

Nutrient starvation is long known to induce *M.tb* into non-replicating persistence [30]. The development of a starvation-induced *in vitro* model of dormancy in *M. tuberculosis* by Loebel [31] has provided a valuable experimental system to screen antidormancy anti-tubercular drugs. We have adopted a variation of this model to test TFP for its lethality against persistent *M.tb* [32]. Mid-log cultures were incubated in phosphate buffered saline (PBS) and left standing at 37°C in sealed flasks. In order to check viability of *M. tuberculosis* during starvation in PBS CFUs from duplicate cultures were assayed at three time points over a 6-week period. As shown in Figure 5, no major loss of viability was observed during this time; with CFU levels remaining almost constant at all three time points. This observation confirmed the attainment of bacteriostasis. Non-replicating bacilli are also known to have a reduced respiration rate. To check whether our model was in agreement with this characteristic of persistent bacilli, methylene blue dye was added to cultures in both nutrient-rich broth and PBS to monitor oxygen consumption. Nutrient rich cultures showed complete dye decolorization and hence, oxygen depletion after 7 days. In contrast, methylene blue dye in the nutrient-starved cultures did not decolorize but remained the same color as the control solution containing no bacteria till 5 weeks. However it showed a slight decrease thereafter till 7th week. This confirmed the decrease in respiration rate of starved *M.tb*. The phenotypic resistance of dormant *M.tb* to various first line drugs is well known. Of these Isoniazid (INH) and Rifampicin resistance has been used as a marker for dormancy. In our studies six-week-starved cultures were treated for 5 days with isoniazid and TFP in duplicate at their respective MICs (0.25 µg/ml for INH and 10 µg/ml for TFP). Figure 4 shows the effect of these treatments on culture viability. The nutrient-starved cultures showed resistance to isoniazid at its MIC for actively growing cultures, with no change in CFUs over a period of 7 days. In contrast the nutrient starved cultures remained sensitive to TFP at its MIC for actively growing cultures, with a decrease in viability by 2.3 log_10_ units as compared to that of INH treated culture. Dissimilarity in response of starved cultures to treatment with TFP and INH illustrate the different mechanism of actions of both the compounds. Since INH is known to target mycolic acid synthesis [33], the phenotypic resistance of persistent *M.tb* to INH relates to the absence of requirement of the cell wall synthesis by non-replicating bacilli. Hence the starved cultures continue to survive well even in presence of INH at its MIC.

### TFP is Effective Against NO Induced NRP *M.tb*

**Figure 6:**
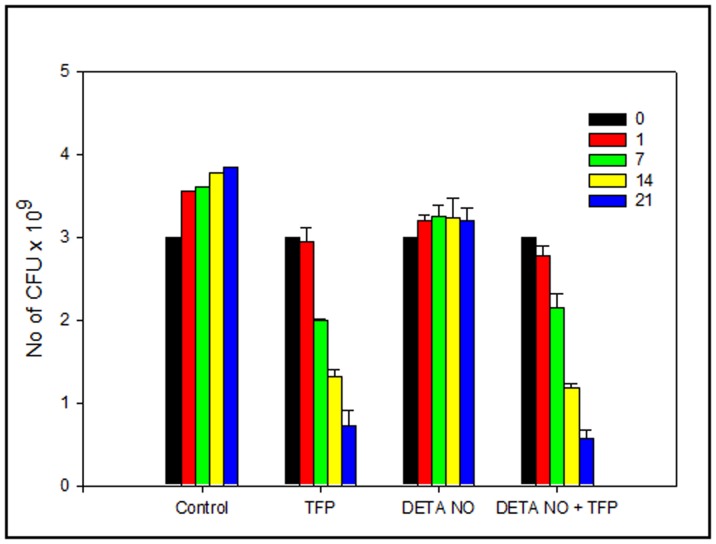
Susceptibility of NO induced NRP *M.tb* cultures to TFP. Mid log phase *M.tb* cultures were subjected to NO stress by treating with 50 uM DETA NO for 16 hrs. Attainment of bacteriostasis was confirmed by enumerating CFU at week 1, 2 and 3. After 16 hr treatment culture was treated TFP (5 ug/ml). Growth kinetics of treated and untreated bacilli in presence of TFP was monitored by enumerating CFU at days 1, 7, 14 and 21 post treatment.

The ability of *M.tb* to persist chronically inside host well beyond the initial acute phase can also be attributed to the activation of the host immune system. There has been compelling evidence that RNI contribute significantly to the control of chronic persistent tuberculous infection [34]. Nitric oxide is known to induce expression of a *M. tuberculosis*–derived 16-kDa heat shock protein, a molecule that promotes stationary phase of growth of mycobacteria, hence the bacilli enter the NRP stage [35]. This in vivo condition has been mimicked in vitro by using a nitric oxide donor like DETA-NO in broth cultures [36]. Such a model can be used to screen compounds that can contribute effectively to eradication of dormant TB. In our present study treatment of mid-log phase *M.tb* culture with 50 uM DETA NO lead to stasis of the culture when checked for a period of three weeks. This transition from active to persistent phase is accompanied by shift of bacilli to an alternative respiratory chain with nitrate serving as an acceptor in absence of oxygen. We have checked the effect of TFP on survival of NO induced NRP bacilli by subjecting DETA NO (50 µM) treated *M.tb* cultures to TFP at a concentration of 5 µg/ml as described in materials and methods. The result is shown in figure 6. The graph clearly shows that after a 16 hr treatment with 50 µM DETA NO, *M.tb* cultures were found to be static showing minimal variation in number of CFUs as checked till 3 weeks post treatment. However the untreated cultures showed a steady growth rate during the same time interval. Effect of TFP at 5 µg/ml was checked on both DETA NO treated and untreated cultures. Presence of TFP showed similar effect on both the cultures showing a decrease of 81 in number of CFU by day 21 as compared to the respective controls. Result shows that this NO induced persistent bacilli is susceptible to TFP wherein TFP was able to significantly reduce the survival of bacilli in the stationary model.

## Discussion

The resistance of *M.tuberculosis* to various stresses has been considered one of the major factors that have led to its success as an intracellular pathogen. This is so because *M.tuberculosis* is located in pulmonary cavities within caseous material where the pH, oxygen and nutrition are sufficiently low. Not only this, the active immune response of host to this pathogen involves release of highly reactive oxygen and nitrogen intermediates, which are toxic to the bacilli. But mycobacterium has developed various strategies to counteract these conditions. Antibiotics used to treat TB infection are usually active against growing bacteria but not against the dormant pathogen. Correlation between antibiotic activity and bacterial growth state in streptomycin-dependent *M.tb* was shown almost 30 years ago. The antibiotic-resistance of non-growing bacteria is due to changes in bacterial metabolism or physiological state and is described as phenotypic resistance. While the dormant bacilli are known to effectively escape the immune system acquiring phenotypic resistance to the current first line drugs, many clinical isolates have been found to have developed genetic level resistance to TB chemotherapy. Hence the need of the hour is development of drugs that can prevent the pathogen from surviving in a drug-resistant state. Such drugs in combination with the current antibiotics can reduce the period of treatment for complete cure and lead to global eradication of TB.

Though many reports have indicated the anti-mycobacterial activity of TFP [5], [7], but its exact mechanism of action is not yet clearly understood. It has already been shown that phenothiazines are effective against MDR *M.tb* making them potential candidates for novel TB chemotherapy [6]. In the present study we have demonstrated the ability of TFP to effectively kill two of the multi drug resistant clinical isolates *M.tb*JAL2287 and *M.tb*1934 *in vitro* as well as *ex vivo.* The activity of compounds in the macrophage model can easily be considered a more accurate reflection of the effect of the complex environment encountered by *M. tuberculosis* during infection, on drug activity. In our study we monitored the effect of TFP on intracellular MDR *M.tb* for a period of 3 days post infection in activated THP-1 cells and human monocyte derived macrophages, where a significant reduction in no. of CFU was observed in presence of TFP. This period would correspond to an active infection stage *in vivo*. TFP being able to accumulate within macrophages is expected to kill *M.tb* during this phase at concentrations that are clinically allowed. Further we wanted to check if this compound is effective in the latent phase of infection. Dormant bacilli are particularly resistant to current first line drugs, because these drugs target processes that are required by actively dividing cells. If a compound is able to kill the persistent bacilli, it can very well be speculated that its targets are critical for dormant bacilli survival. With this view in mind we tested the effect of TFP on survival of stress induced persistent bacilli. TFP was able to inhibit the survival of bacilli under all three conditions of stress – acidic, starvation and presence of nitric oxide. This inhibition clearly shows that TFP’s targets in *M.tb* are required by the bacilli for persistence. This is further supported by the fact that the two of the mycobacterial enzymes namely type II NADH oxidoreductase (NDH-2) and malonyl coenzyme A: acyl carrier protein transacylase (MCAT), shown to be inhibited by TFP, seem to be required for the survival of *M.tb* during starvation. NDH-2 is required to sustain ATP homeostasis during dormancy [37] whereas MCAT is involved in fatty acid synthesis [38], which is the major nutrient source for *M.tb* during latency. Also since TFP acts as an antagonist to *M.tb* CAMLP, which is itself an activator protein, it can be said that all its downstream targets will be indirectly inhibited by TFP. However, it still remains to be seen as to what all processes are mediated by CAMLP in *M.tb* and whether these are required or differentially regulated during dormancy.

Individuals with TB infection are known to possess heterogeneous populations presumably living in active and latent TB lesions. Our study shows the ability of TFP to kill both of these populations indicating that it targets pathways that are common to both active and various stress induced dormant infection. Monotherapy of TB is known to be the cause for the development of drug resistance, therefore multidrug therapy has been recommended for TB. In order to combat a tough infection like TB, a number of approaches should be used in testing for antimicrobial susceptibility, so as to facilitate further *in vivo* experimentation with drug combinations. Synergistic interaction of phenothiazines with a wide spectrum of antimicrobial agents including conventional TB drugs has been shown [39]. Previously TFP has also shown the potential to enhance the accumulation and retention of other anti-mycobacterial drugs in macrophages [40]. Thus, following further evaluation these compounds can be used as an adjunct to current regimens for the management of TB. Also development of more effective and less toxic derivatives of TFP can be an alternative approach to harness the functions of the compound.
